# Supplementary material for: A common mechanism of proteasome impairment by neurodegenerative disease-associated oligomers
Source: Nat Commun. 2018 Mar 15;9:1097. doi: 10.1038/s41467-018-03509-0 (PMC5854577; doi:10.1038/s41467-018-03509-0)
Supplement: Supplementary file 1 — Supplementary Information(PDF 683 kb) [file 41467_2018_3509_MOESM1_ESM.pdf]

A COMMON MECHANISM OF PROTEASOME IMPAIRMENT BY NEURODEGENERATIVE DISEASE-ASSOCIATED OLIGOMERS

Supplementary Information
Figures 1-10

Thibaudeau et al.

*Correspondence to
David M. Smith
dmsmith@hsc.wvu.edu

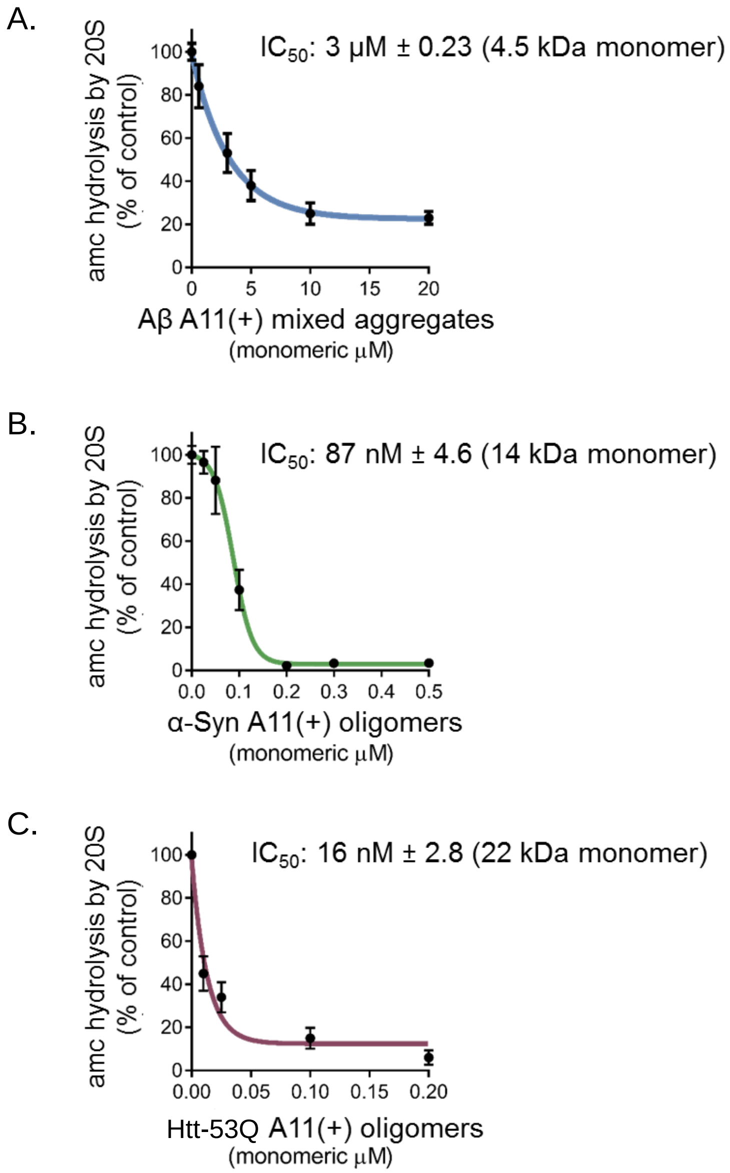

Corresponds to Figure 1B.

Supplementary Figure 1. A11(+) soluble aggregates of Aβ, α-Syn, and Htt-53Q impair the mammalian 20S proteasome in a concentration-dependent manner. Mammalian 20S proteasomes were incubated with soluble aggregates of Aβ (A), α-Syn (B), or Htt-53Q (C) at the indicated concentrations and proteasome activity (LLVY-amc hydrolysis) was measured. Aβ mixed aggregates are the same as used in Figure 1A. Soluble oligomers from α-Syn and Htt-53Q are from Figure 1B. Half-maximal inhibition of the 20S is indicated. The concentrations of aggregates are calculated based on the respective monomeric peptide/protein mass (Aβ, 4.5 kDa; α-Syn, 14 kDa; and Htt-53Q, 22 kDa). All controls contained an equal volume of buffer identical to that of the respective aggregates. Data is representative of three independent experiments performed in triplicate. Error bars represent ± standard deviation.

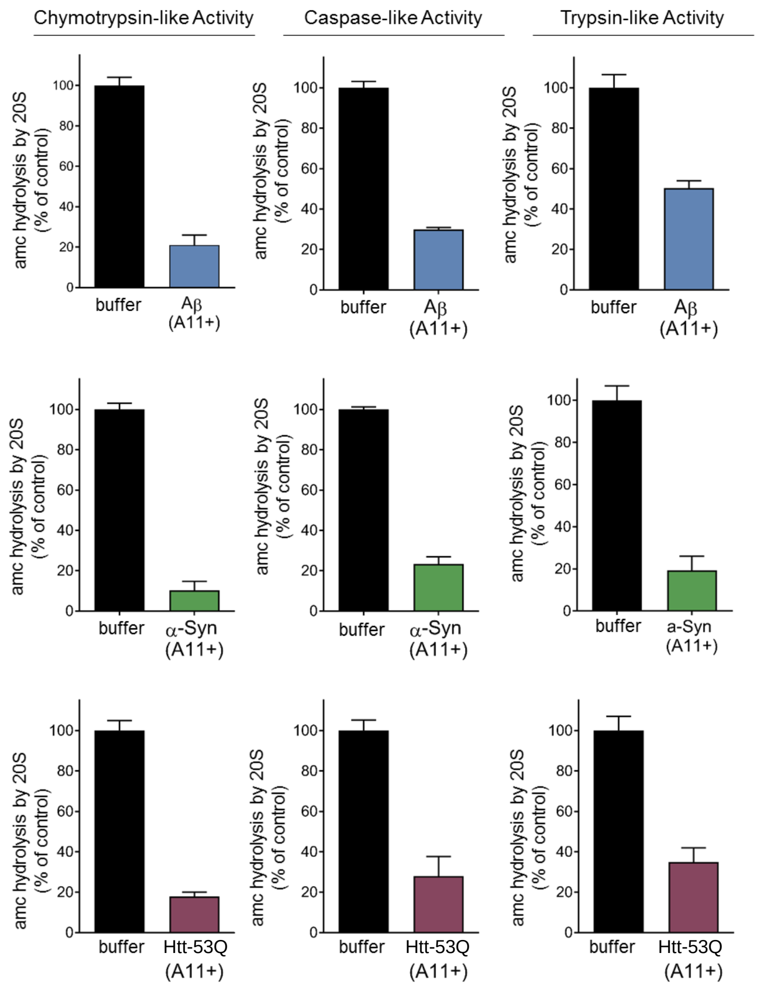

Corresponds to Figure 1B.

Supplementary Figure 2. A11(+) oligomers from Aβ, α-Syn, and Htt-53Q impair substrates that are specific for all three proteolytic sites in the mammalian 20S proteasome. Mammalian 20S proteasomes were incubated with Aβ oligomers (2μM) (***A***), α-Syn A11+ oligomers (1μM) (***B***), or Htt-53Q A11+ oligomers (0.1μM) (***C***), and proteasome chymotrypsin-like (LLVY-amc hydrolysis), caspase-like (nLPnLD-amc hydrolysis), and trypsin-like (RLR-amc hydrolysis) activity was measured. Chymotrypsin-like and caspase-like activity assays used 0.5nM of 20S proteasome, trypsin-like activity assays used 1nM of 20S proteasome. The concentrations of aggregates are calculated based on the respective monomeric peptide/protein mass (Aβ, 4.5 kDa; α-Syn, 14 kDa; and Htt-53Q, 22 kDa). All controls contained an equal volume of buffer identical to that of the respective aggregates. Data is representative of three independent experiments performed in triplicate. Error bars represent ± standard deviation.

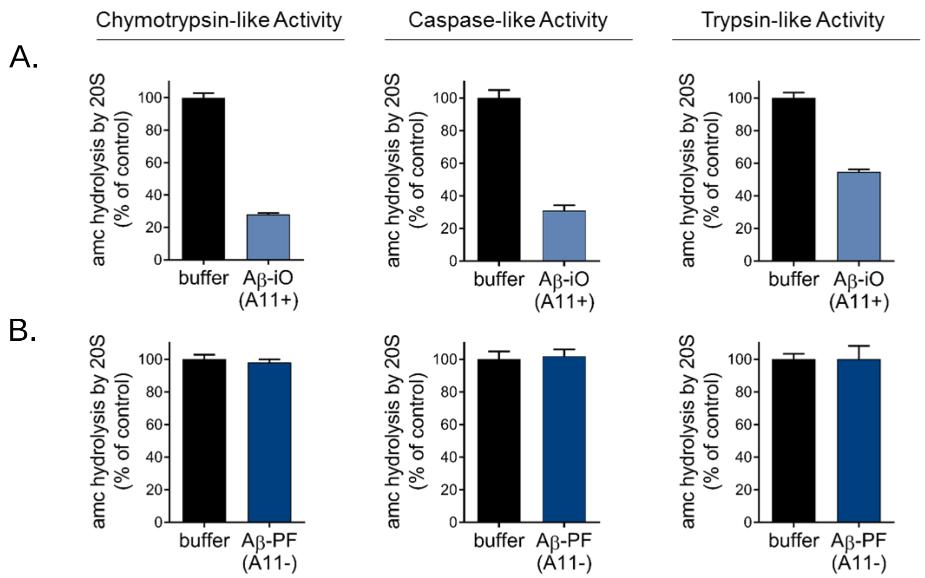

Corresponds to Figure 1C.

Supplementary Figure 3. A11(+) Aβ intermediate oligomers (Aβ-iO) impair the degradation of substrates specific for each of the three peptidase activities in the 20S, and A11(-) Aβ protofibrils (Aβ-PF) do not have any effect. Mammalian 20S proteasomes were incubated with peak Aβ fractions from size exclusion chromatography in Fig. 1*D:* 2.5μM Aβ-iO (***A***) or 5μM Aβ-PF (***B***). Chymotrypsin-like (LLVY-amc hydrolysis, rfu/min) and caspase-like (nLPnLD-amc hydrolysis, rfu/min) activity assays used 0.5nM of 20S proteasome, trypsin-like (RLR-amc hydrolysis, rfu/min) activity assays used 2nM of 20S proteasome. The concentrations of aggregates are calculated based on the monomeric peptide mass (4.5 kDa). All controls contained an equal volume of buffer identical to that of the respective aggregates. Data is representative of three independent experiments performed in triplicate. Error bars represent ± standard deviation.

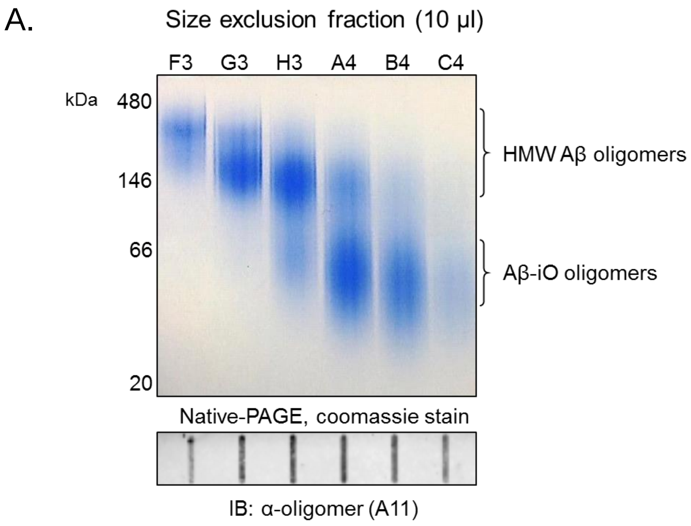

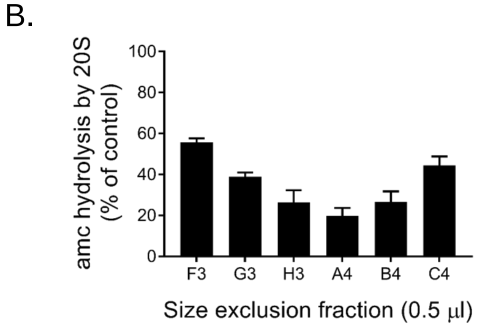

Corresponds to Figure 1C.

Supplementary Figure 4. Soluble high molecular weight Aβ oligomers also impair the 20S proteasome if they are A11 positive. (***A***) High molecular weight A11(+) oligomers of Aβ were separated by size exclusion chromatography, peak fractions (10µl) were separated by native-PAGE and coomassie stained (top). Each fraction (2µl) was evaluated by slot blot for A11 immunoreactivity (bottom). (***B***) 20S proteasome activity (nLPnLD-amc hydrolysis, rfu/min) was determined with fractions from part A (0.5µl). The concentrations of aggregates are calculated based on the monomeric peptide mass (4.5 kDa). All controls contained an equal volume of buffer identical to that of the respective aggregates. Data is representative of three independent experiments performed in triplicate. Error bars represent ± standard deviation.

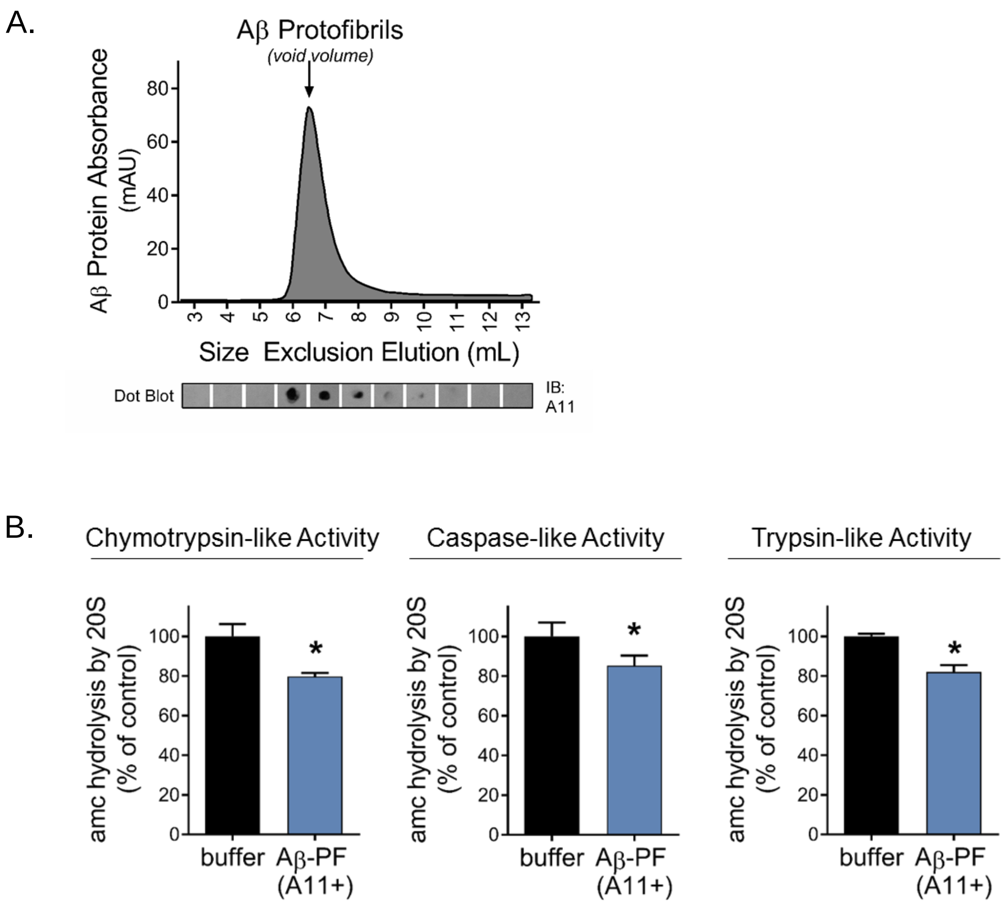

Corresponds to Figure 1C.

Supplementary Figure 5. A11(+) Aβ protofibrils impair the mammalian 20S proteasome. (***A***) A11(+) Aβ protofibril preparation was separated by size exclusion chromatography (Superose 6 GL). The protofibrils eluted in the column void volume, indicating the soluble aggregates are >700 kDa (top). Equal volumes of each fraction were probed for A11 reactivity (bottom). (***B***) Mammalian proteasomes were incubated with A11(+) Aβ protofibrils (2.5μM) and the activity of all three active sites was measured by fluorescent substrate hydrolysis. The concentrations of aggregates are calculated based on the monomeric peptide mass (4.5 kDa). All controls contained an equal volume of buffer identical to that of the respective aggregates. Data is representative of three independent experiments performed in triplicate. Error bars represent ± standard deviation. * = $p < 0.05$.

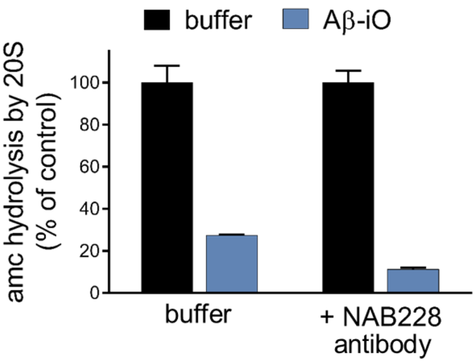

Corresponds to Figure 1E.

Supplementary Figure 6. Incubation with antibody targeted to the Aβ N-terminus (1-8) does not rescue proteasome activity. Aβ intermediate oligomers (Aβ-iO) from Fig. *1D* were incubated with buffer or monoclonal antibody (NAB228, Invitrogen, epitope 1-8 Aβ peptide) for 30 minutes at 37°C and tested for effect on proteasome activity (nLPnLD-amc hydrolysis). The concentration of oligomers are calculated based on the monomeric peptide mass (4.5 kDa). Controls contained an equal volume of buffer identical to that of the Aβ oligomers and/or the NAB228 antibody. Data is representative of three independent experiments performed in triplicate. Error bars represent ± standard deviation.

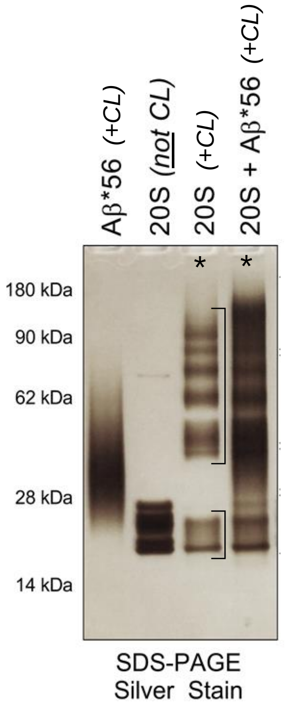

Corresponds to Figure 3A.

Supplementary Figure 7. Aβ*56 oligomers bind to the 20S proteasome. (**A**) 20S proteasomes (0.4μg) and Aβ*56 oligomers (1.5μg) (from Figure 3A) were incubated separately or together for 30 minutes (37°C), were lightly crosslinked for 5min, and then separated by SDS-PAGE (4-12% bis-tris gel). Total protein was detected by silver stain. Lane 2 shows 20S proteasome migration pattern without crosslinking for comparison. Lane 3 brackets denote intra-proteasome crosslinked subunits after glutaraldehyde treatment (top bracket) and individual subunits that did not crosslink (bottom bracket). The lack of aggregated protein in the stacking gel (*) indicating minimal crosslinking conditions.

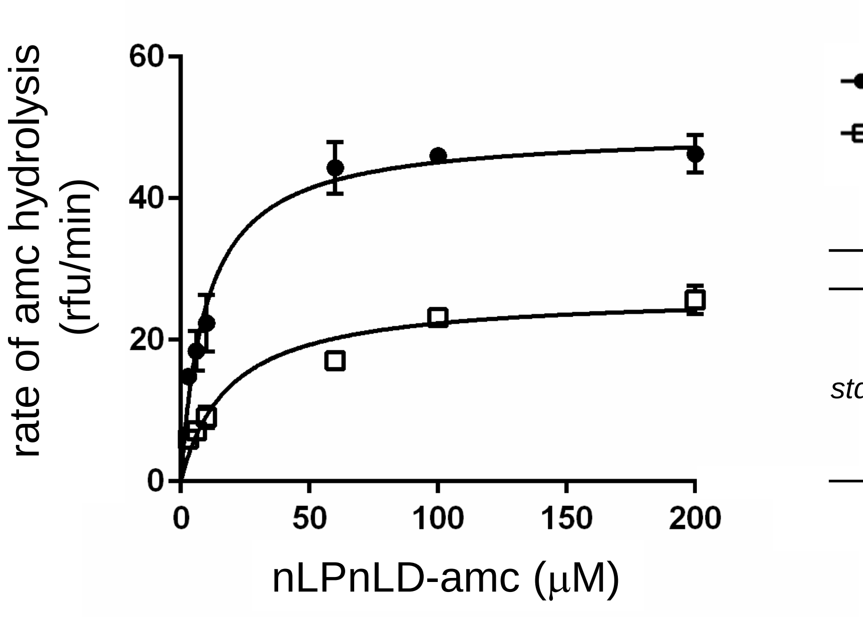

	buffer	Ab*56
V_{max}	49.51	26.48
K_m	9.816	18.57
std. error		
V_{max}	*1.568*	*1.238*
K_m	*1.183*	*3.355*

Corresponds to Figure 3.

Supplementary Figure 8. Substrate saturation curve on the WT 20S proteasome with and without Aβ*56 oligomers. Mammalian 20S proteasomes (0.5nM) were assayed for nLPnLD-amc hydrolysis activity with and without Aβ*56 oligomers (1.2μM) for 60 minutes at various substrate concentrations. The rate of amc-hydrolysis was plotted and line fit using non-linear regression and the Michaelis-Menten equation (GraphPad). V_{max} and K_m values are shown to the right. Experiment was performed in triplicate.

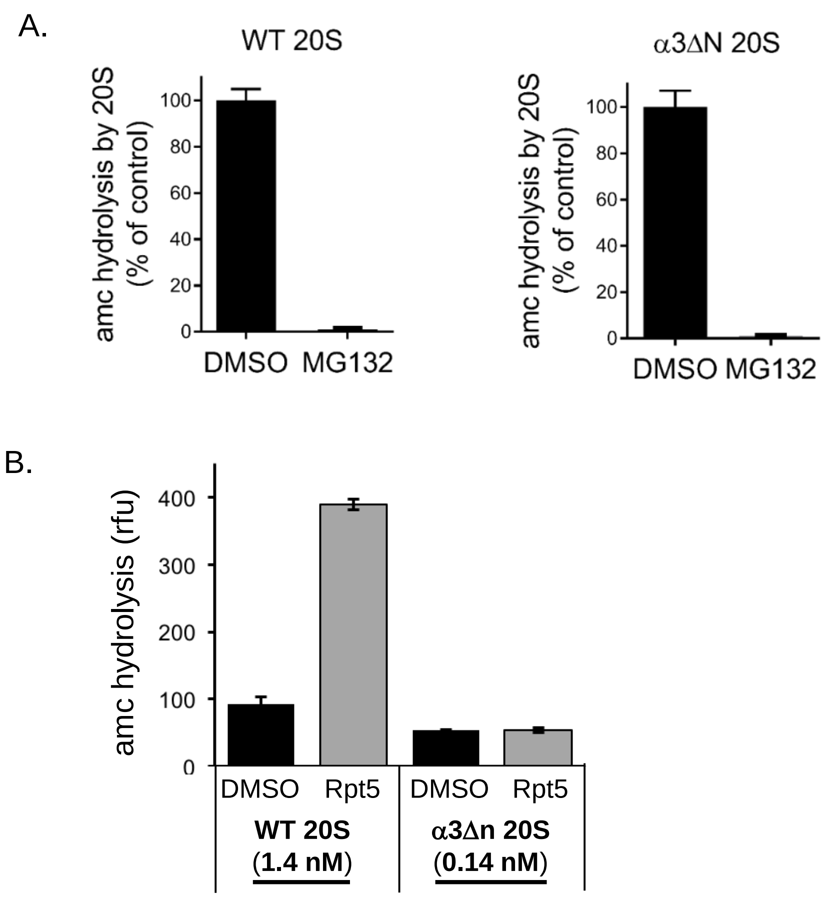

Corresponds to Figure 3B-D.

Supplementaary Figure 9. WT and α3ΔN proteasome preparations have expected activities. (***A***) Chymotrypsin-like activity (LLVY-amc hydrolysis) is abolished in both wild-type 20S and α3ΔN 20S mutant proteasomes after pre-treatment with proteasome inhibitor MG132 (50μM). (***B***) Wild type 20S proteasomes (1.4 nM) show gate opening by addition of a known gate-opening peptide, Rpt 5 (300μM). However, as expected the Rpt5 peptide could not stimulate the open-gate mutant α3ΔN 20S proteasomes (0.14 nM). Note there is 10X more WT 20S than α3ΔN 20S. Data is representative of three independent experiments performed in triplicate. Error bars represent ± standard deviation.

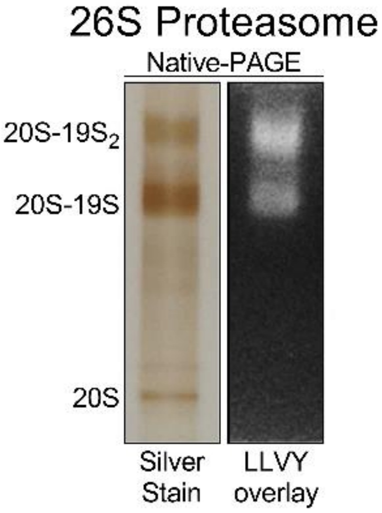

Corresponds to Figure 6A.

Supplementary figure 10. **The measured amc hydrolysis from 26S proteasome preparation is due to single and double capped 26S activity and not 20S activity**. Native-PAGE of 1µg of mammalian rabbit muscle 26S proteasome. In-gel enzyme activity assay (LLVY-amc hydrolysis) (left), silver stain (right). Gel is representative of three 26S proteasome preparations from rabbit muscle. Note that the LLVY-amc activity is only detectable from the 20S-19S complexes in these experiments.
